# Systematic analysis of the human tumor cell binding to human vs. murine E- and P-selectin under static vs. dynamic conditions

**DOI:** 10.1093/glycob/cwaa019

**Published:** 2020-02-27

**Authors:** Sarah Starzonek, Hanna Maar, Vera Labitzky, Daniel Wicklein, Charlotte Rossdam, Falk F R Buettner, Gerrit Wolters-Eisfeld, Cenap Guengoer, Christoph Wagener, Udo Schumacher, Tobias Lange

**Affiliations:** 2 Institute of Anatomy and Experimental Morphology, University Medical Center Hamburg-Eppendorf, Martinistrasse 52, 20246 Hamburg, Germany; 3 Institute of Clinical Biochemistry, Hannover Medical School, Carl-Neuberg-Str. 1, 30625 Hannover, Germany; 4 Research Institute Children's Cancer Center and Department of Pediatric Hematology and Oncology, University Medical Center Hamburg-Eppendorf, Martinistrasse 52, 20246 Hamburg, German; 5 Department for General, Visceral and Thoracic Surgery, University Medical Center Hamburg-Eppendorf, Martinistrasse 52, 20246 Hamburg, Germany; 6 Center for Diagnostics, University Medical Center Hamburg-Eppendorf, Martinistrasse 52, 20246 Hamburg, Germany

**Keywords:** adhesion, carbohydrate-binding protein, metastasis, shear stress, tumor cell biology

## Abstract

Endothelial E- and P-selectins promote metastasis formation by interacting with sialyl-Lewis X and A (sLeX/sLeA) on circulating tumor cells. This interaction precedes extravasation and can take place under dynamic and static conditions. Metastasis formation is often studied in xenograft models. However, it is unclear whether species differences exist in the ligand specificity of human (h) vs. murine (m) selectins and whether different ligands are functional under dynamic vs. static conditions. We systematically compared the h vs. m E- and P-selectin (ESel/PSel) binding of a range of human tumor cells under dynamic vs. static conditions. The tumor cells were categorized by their sLeA/X status (sLeA+/sLeX+, sLeA−/sLeX+ and sLeA−/sLeX−). The general biological nature of the tumor–selectin interaction was analyzed by applying several tumor cell treatments (anti-sLeA/X blockade, neuraminidase, pronase and inhibition of *O*/*N*-glycosylation). We observed remarkable differences in the static vs. dynamic interaction of tumor cells with h vs. m ESel/PSel depending on their sLeA/X status. The tumor cell treatments mostly affected either static or dynamic as well as either h- or m-selectin interaction. mESel showed a higher diversity of potential ligands than hESel. Inhibition of *O*-GalNAc-glycosylation also affected glycosphingolipid synthesis. Summarized, different ligands on human tumor cells are functional under static vs. dynamic conditions and for the interaction with human vs. murine ESel/PSel. Non-canonical selectin ligands lacking the sLeA/X glycan epitopes exist on human tumor cells. These findings have important implications for the current development of glycomimetic, antimetastatic drugs and encourage the development of immunodeficient mice with humanized selectins.

## Introduction

Altered glycosylation in general and the selectin family of cell-adhesion molecules in particular have attracted considerable attention in cancer and metastasis research over the past decades (Mereiter, Balmana, et al. 2019; Pinho and Reis 2015). All selectin family members contain an extracellular Ca^2+^-dependent (C-type) lectin domain, followed by an epidermal growth factor (EGF)-like motif, a transmembrane domain and a short cytoplasmic tail (Cummings and Smith 1992; Bevilacqua and Nelson 1993). With their C-type lectin domain, selectins bind to carbohydrate ligands on neighboring cells and by this interaction they mediate cell–cell contact (McEver 2002). The binding of E- and P-selectins, expressed on the luminal surface of endothelial cells, to terminal glycan residues including α2,3-linked sialic acid and α1,3/α1,4-linked fucose, expressed on circulating tumor cells (CTCs) (Varki et al. 2009), initiates the adhesion of CTCs to endothelial cells lining the vasculature at a future metastatic site. This initial adhesion crucially promotes metastasis formation (Reymond et al. 2013).

Accordingly, the expression of the canonical selectin ligands, i.e., sialyl-Lewis X (sLeX, CD15s: Neu5Acα2,3Galβ1,4(Fucα1,3)GlcNAc-R) and sialyl-Lewis A (sLeA, CA 19-9: Neu5Acα2,3Galβ1,3(Fuc-α1,4)GlcNAc-R), correlates with metastasis formation and poor outcome of patients suffering from different tumor types such as gastrointestinal cancers (Varki et al. 2009). Likewise, expression levels of the scaffold structures carrying sLeA and sLeX (which can be *N*- or *O*-glycosylated glycoproteins or glycolipids) and of the glycosyltransferases synthesizing sLeA and/or sLeX were also found to correlate with unfavorable outcome in breast cancer patients (Esposito et al. 2019). In vivo experiments using human cells in immunodeficient mice demonstrated that genetic knockout of *Sele* and *Selp* (encoding E- and P-selectins) drastically reduces the number of spontaneous metastases (Köhler et al. 2010; Stübke et al. 2012; Gebauer et al. 2013; Wicklein et al. 2013; Heidemann et al. 2014). Meanwhile, several glycomimetic drugs have been developed that are meant to block selectin–ligand interaction during metastasis and recent publications support upcoming clinical trials (Bull et al. 2015; Esposito et al. 2019).

Most of our current knowledge on selectin–ligand interaction in vivo was obtained using xenograft models, in which human tumor cells were engrafted into immunodeficient mice. However, it is still largely unknown whether species-specific differences exist in the tumor cells’ ligands for human vs. murine E- and P-selectins. Furthermore, the selectin–ligand interaction might not only take place under dynamic conditions (enabling active adhesion of flowing CTCs as described above) but also under static conditions (enabling selectin binding after mechanical trapping of CTCs). Both modalities have been discussed to take place at sites with different microvessel diameters (Sahai 2007; Chaffer and Weinberg 2011; Reymond et al. 2013). However, it is not clear yet whether the same or different selectin ligands are functional under static vs. dynamic conditions.

We therefore investigated whether human tumor cells use different ligands for human vs. murine E- and P-selectins under dynamic adhesion vs. static binding conditions. We systematically analyzed the putative differences in three different groups of human tumor cells, which were categorized by the presence or absence of sLeA and/or sLeX. Moreover, we examined the functional relevance of terminal sialic acid, cell surface glycoproteins as well as glycoprotein-bound *N*- vs. *O*-glycans for human vs. murine E- and P-selectin dynamic adhesion vs. static binding. By this, we aimed to analyze whether global patterns of the tumor cell–selectin interaction exist among the chosen tumor cell categories.

## Results

We compared nine human malignant cell lines for their static binding vs*.* dynamic adhesion behavior to recombinant human vs. murine E- and P-selectins (hESel, hPSel, mESel and mPSel). The tumor cell lines were grouped depending on their cell surface selectin ligand status. HT29, PaCa5061 and GC5023 cells expressed both canonical ligands (group I: sLeA/X-positive). The cell lines EOL-1, DU4475 and Molm13 expressed sLeX only (group II: sLeX-positive). HOS, MV3 and SKOV3 cells lacked both sialyl-Lewis antigens (group III: sLeA/X-negative).

### Static binding of human vs. murine E- and P-selectins by human tumor cells with different sLeA and sLeX status

**Fig. 1:**
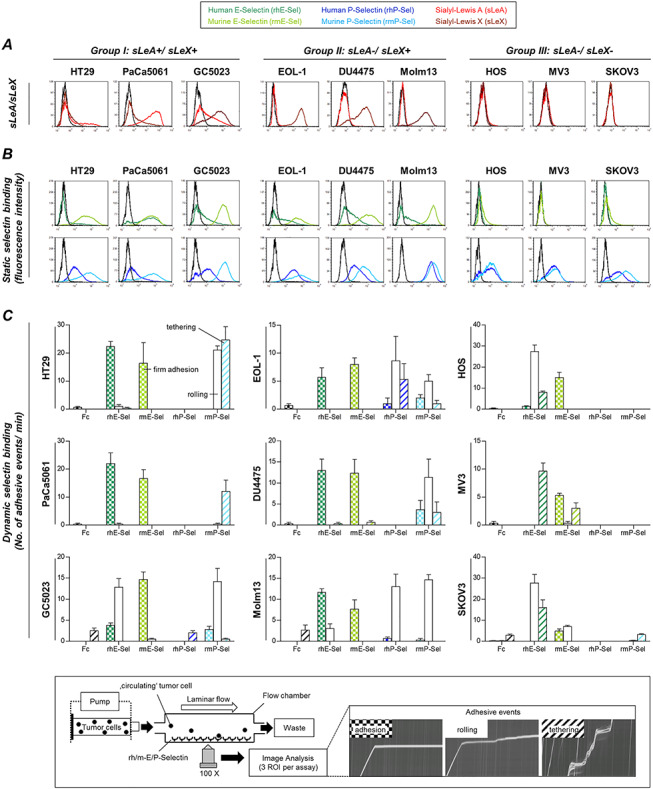
Human tumor cells categorized for their sialyl-Lewis A and X (sLeA/X) status show divergent static binding vs*.* dynamic adhesion to human vs*.* murine E- and P-selectins. sLeA/X expression (**A**) and static binding of selectins (**B**) were analyzed by flow cytometry (black curves represent control/isotype conditions). Dynamic adhesion on selectins (**C**) was tested in laminar flow adhesion experiments as illustrated in the insert. Adhesive events were distinguished into firm adhesion, rolling and tethering. Please note the color code legend above panel (**A**). Bars in (**C**) represent mean ± SD of triplicate recordings each from two independent experiments. Black bars represent nonspecific binding to IgG-Fc control. Briefly, sLeA and/or sLeX are apparently required for static E-selectin binding (**A** and **B**) and dynamic adhesion on mPSel (**C**). sLeA/X-negative cells developed loose dynamic adhesions on hESel and firm adhesions on mESel (**C**) and were able to bind P-selectins under static conditions (**B**). The only cell lines with notable dynamic adhesions on hPSel (**C**) were both derived from leukemia patients (EOL-1 and Molm13) and were the only ones that expressed PSGL-1 (Supplementary Figure 1). mESel was commonly more strongly bound than hESel (**B** [all groups] + **C** [group III]). This figure is available in black and white in print and in colour at *Glycobiology* online.

The sLeA and sLeX status of the tested cells is shown in Figure 1A. All cells were capable of binding hPSel and mPSel (Figure 1B). There were only marginal species differences in the binding capacity for P-selectin in the sLeX-positive or sLeA/X-negative group. However, the sLeA/X-positive group showed considerably more murine than human P-selectin binding (Figure 1B). hESel and mESel binding was observable in the sLeA/X- or sLeX-positive groups, while the cells commonly bound much more mESel than hESel (Figure 1B). Within the sLeA/X-negative group, HOS and SKOV3 cells showed very weak levels of mESel binding (Figure 1B), but none of them showed static hESel binding.

### Dynamic adhesion of human tumor cells with different sLeA and sLeX status on human vs. murine E- and P-selectins

In contrast, all tested cell lines were capable of forming dynamic adhesions on hESel (even those of the sLeA/X-negative group that were unable to bind hESel under static conditions); however, the quality of this interaction was notably different: firm adhesions were observed in case of the sLeA/X- or sLeX-positive group, while loose adhesions (rolling and/or tethering) were observed in case of the sLeA/X-negative group (Figure 1C). In contrast, all cell lines developed firm adhesions on mEsel irrespective of the sLeA/X status (Figure 1C). Dynamic adhesion on mPSel was only observed in the sLeA/X- or sLeX-positive groups (Figure 1C). Among all tested cell lines, only EOL-1 and Molm13 (both myeloid leukemia cell lines, second group) formed dynamic adhesions on hPSel (Figure 1C). These two cell lines were the only ones with PSGL-1 expression (Supplementary Figure 1A). Blocking PSGL-1 on EOL-1 and Molm13 cells notably reduced static hPSel binding (−64% and −71%, respectively), while mPSel binding was impaired much less (−14% and −32%, respectively). This species-specific difference was also observed in the dynamic adhesion experiments, where the PSGL-1 blockade caused a switch from firmer (rolling) to looser (tethering) interactions on hPSel while adhesion on mPSel was not affected (Supplementary Figure 1B). See Supplementary Table I for an overview on selectin binding capacity of the cell line groups I–III.

### Glycospecificity of murine vs. human E-selectin in a direct binding assay

**Fig. 2:**
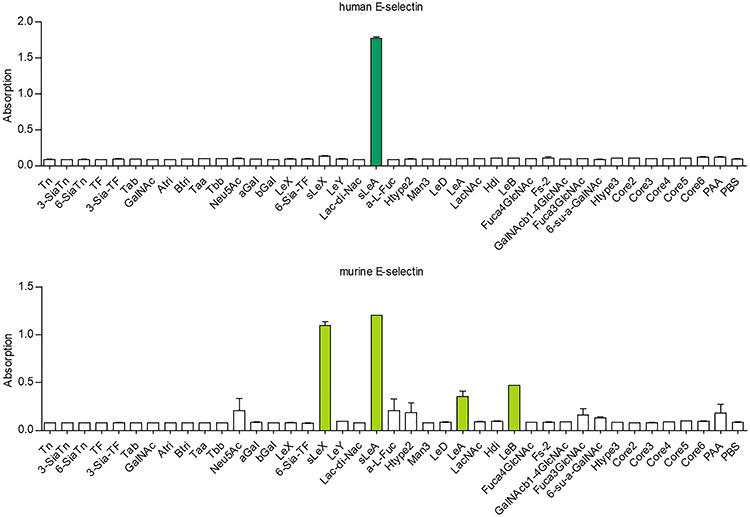
Carbohydrate specificity of human vs. murine E-selectin. Direct E-selectin glycan binding assay of streptavidin-HRP complexed human vs. murine E-selectin on a 96-well plate coated with the annotated glycans. Note the broader carbohydrate binding pattern of murine E-selectin. Bars represent mean ± SEM of two technical replicates. This figure is available in black and white in print and in colour at *Glycobiology* online.

The glycan-binding specificities of human and murine E-selectin were examined in a direct binding study with various glycoconjugates that included core and tumor-associated *O*-glycans, *N*-glycan-specific structures, ABO blood groups, monosaccharides, Lewis structures and controls. As shown in Figure 2, hESel displayed a specific and unique binding to sLeA, while mESel bound sLeA, sLeX, Lewis A (LeA) and Lewis B (LeB).

### sLeA and/or sLeX blockade experiments with sLeA/X-positive tumor cells

As shown in Supplementary Figure 2A, static hESel binding of the sLeA/X-positive group could be largely blocked by anti-sLeA (−69.3% to −96.8%), but only slightly reduced by anti-sLeX (−22.4%). Only in case of GC5023 cells, the combination of sLeA and sLeX blockade cooperatively reduced hESel binding (−92.4%). In contrast, mESel binding was largely unaffected by sLeA and/or sLeX blockade while the effects on h/mPSel binding differed among the tested cell lines (see Supplementary Figure 2A). hPSel binding commonly increased by 6.2–53% after sLeA and/or sLeX blockade.

In the dynamic adhesion experiments (Supplementary Figure 2B), we observed almost no change upon sLeA and/or sLeX blockade except decreased firm adhesions of GC5023 cells on hESel after sLeA blockade and increased tethering events of PaCa5061 cells on mPSel. After combined sLeA/X blockade, we found decreased firm adhesions of PaCa5061 cells on hESel and increased rolling events of GC5023 on mESel. GC5023 cells showed an almost significant reduction of firm adhesions on hESel after sLeX blockade (*P* = 0.055).

### sLeX blockade experiments with sLeX-positive tumor cells

In case of the sLeX-positive group (Supplementary Figure 2C and D), the sLeX blockade reduced static hESel binding (−73.3% to −92.2%), while mESel binding was again largely unaffected except for Molm13 cells showing a 33% reduction in mESel binding. Effects on h/mPSel binding were again less striking and differed among the cell lines. Both myeloid leukemia cell lines (EOL-1, Molm13) showed a 35% reduction in mPSel binding upon sLeX blockade while hPSel binding was increased by trend (Supplementary Figure 2C). Under dynamic conditions, the sLeX blockade reduced the adhesion of DU4475 breast cancer cells on hESel while all other assays remained unaltered (Supplementary Figure 2D).

### Effects of neuraminidase treatment on tumor–selectin interaction

**Fig. 3:**
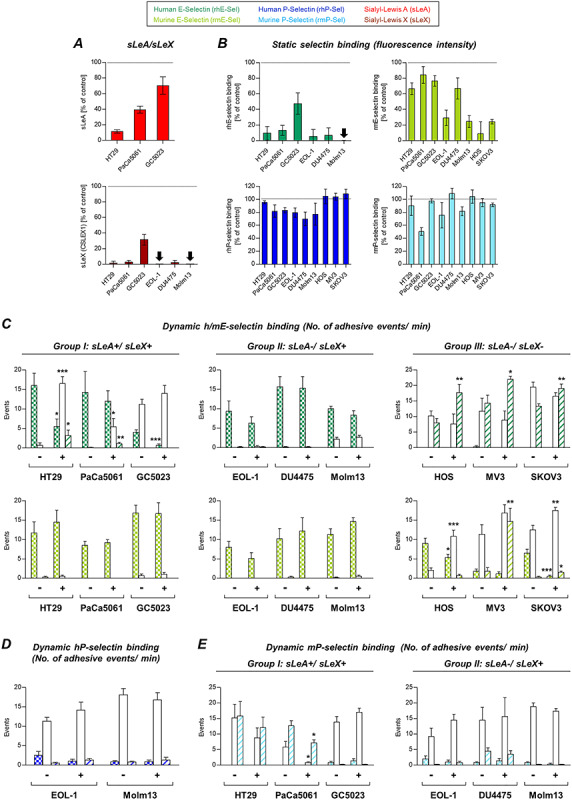
Role of sialic acid residues for tumor cell–selectin interaction. Effects of enzymatic cleavage of terminal sialic acid residues using neuraminidase (*V. cholerae*) on sLeA/X expression and static selectin binding are shown in (**A** and **B**), respectively. The effects of this treatment on the dynamic adhesion of tumor cells on hESel and mESel (**C**), hPSel (**D**) and mPSel (**E**) varied among the tumor cells. Importantly, note the species-specific differences in the efficacy of neuraminidase on static E-selectin binding (**B**). Despite abrogated static hESel binding (**B**), most of the tested cell lines still developed dynamic adhesions on hESel (**C**). Bars in (**A** and **B**) represent mean ± SD of changes of fluorescence intensity relative to controls (represented by the black dotted lines, biological triplicates). Bars in (**C**–**E**) represent means ± SD of triplicate recordings each from two independent experiments; ^*^P ≤ 0.05, ^**^P ≤ 0.01 and ^***^P ≤ 0.001; comparisons were made between treated (+) vs. control (−) cells within the subsets of different adhesive interactions (firm, rolling or tethering adhesion). This figure is available in black and white in print and in colour at *Glycobiology* online.

*Vibrio cholerae* neuraminidase treatment (cleaving terminal sialic acid residues from the tumor cell surface) had varying effects on sLeA expression (~90% reduction in HT29 but only 60% and 25% reduction on PaCa5061 and GC5023 cells, respectively), while sLeX was commonly sharply reduced, except on GC5023 cells (−70% only, Figure 3A). sLeA/X expression was measured by using monoclonal antibodies. Static hESel binding strikingly decreased (90–95%) after neuraminidase treatment with an exception for GC5023 cells (−50% only) (Figure 3B). mESel binding was reduced by 20–40% only in case of HT29, PaCa5061, GC5023 and DU4475 cells, while EOL-1 and Molm13 cells showed ~75% decrease in static mESel binding. The faintly detectable mESel binding of two of the sLeA/X-negative cells (HOS and SKOV3) was also markedly impaired by neuraminidase treatment (Figure 3B). Static hPSel and mPSel binding was only slightly decreased in some cell lines (strongest effect: 50% reduction of mPSel binding by PaCa5061 cells, Figure 3B).

In the dynamic adhesion experiments, neuraminidase treatment reduced firm adhesions on hESel only in case of HT29 and GC5023 cells. All other cell lines remained unaltered or showed an increase of loose adhesions on hESel. Dynamic adhesion on mESel was only affected in the sLeA/X-negative group (Figure 3C), while adhesion on hPSel remained unaltered (Figure 3D). Only PaCa5061 cells showed reduced adhesion on mPSel under flow after neuraminidase treatment (Figure 3E).

### Effects of GalNAc-α-*O*-benzyl treatment on tumor–selectin interaction

**Fig. 4:**
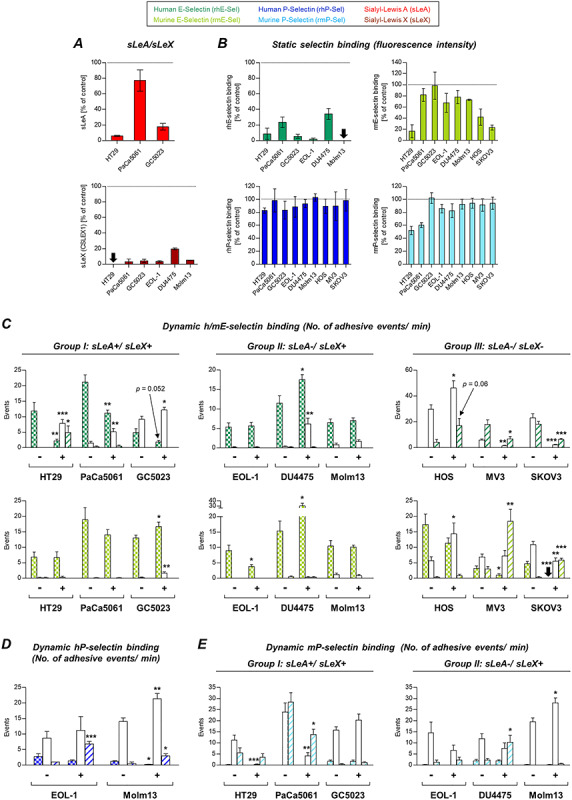
Effects of GalNAc-α-*O*-benzyl treatment on tumor cell–selectin interaction. Expression of sLeA on HT29 and GC5023 as well as sLeX on all sLeX-expressing cells was strongly decreased after treatment with GalNAc-α-*O*-benzyl (**A**). Static binding to hESel was reduced by more than 65% for all tested cell lines, while strong effects on mESel binding (>50% reduction) were only observable for HT29 and the two sLeA/X-negative cells lines HOS and SKOV3 (**B**). P-selectin binding remained unaffected except mPSel binding by HT29 and PaCa5061 cells (**B**). The strongest effects on dynamic adhesions were seen for sLeA/X-positive cells on hESel and sLeA/X-negative cells on hESel and mESel (**C**). Effects on adhesion to P-selectins under flow conditions were less striking and differed among the cell lines (**D** and **E**). Note the discrepant effects of GalNAc-α-*O*-benzyl on static vs. dynamic hESel and mESel interaction (sLeX-positive group); in case of mPSel, however, significant reductions of static binding were also visible in the dynamic experiment (HT29 and PaCa5061). See legend to Figure 3 for technical information. This figure is available in black and white in print and in colour at *Glycobiology* online.

GalNAc-α-*O*-benzyl treatment (impairing *O*-GalNAc-glycosylation) nearly abrogated sLeA and sLeX expressions on tumor cells except sLeA on PaCa5061 cells (Figure 4A) and also commonly and drastically reduced static hESel binding (Figure 4B). In contrast, static mESel binding was only reduced in case of HT29, HOS and SKOV3 cells, while it was largely unaltered for the other cell lines (Figure 4B). Static hPSel binding was almost unaffected except a 20% reduction in case of HT29 cells, while mPSel binding was impaired by 50–60% in case of HT29 and PaCa5061 cells (Figure 4B).

The effects of GalNAc-α-*O*-benzyl treatment on the dynamic adhesion of tumor cells on selectins are summarized in Figure 4C–E. Most strikingly, this treatment reduced the number of firm adhesions on hESel developed by all cell lines of the sLeA/X-positive group (*P* = 0.052 in case of GC5023 cells); vice versa, the number of looser adhesions increased. In the sLeA/X-negative group, two of the tested cell lines (MV3 and SKOV3) showed a decrease of the rolling events on hESel. In this case, the looser interactions (tethering) were decreased as well. DU4475 and HOS cells showed increased adhesions on hESel upon GalNAc-α-*O*-benzyl (Figure 4C). Dynamic adhesion on mESel was variably affected. The firm adhesions of EOL-1, MV3 and SKOV3 cells were reduced, while they increased in case of GC5023 and DU4475 cells (Figure 4C). Effects on dynamic adhesion on hPSel were mainly visible in form of more loose interactions (Figure 4D). The adhesion on mPSel was strongly impaired in case of HT29 and PaCa5061 (but not GC5023) cells; DU4475 cells showed an increase in the weaker tethering interaction, and Molm13 showed an increase in the firmer rolling interaction (Figure 4E).

### Effects of pronase treatment on tumor–selectin interaction

**Fig. 5:**
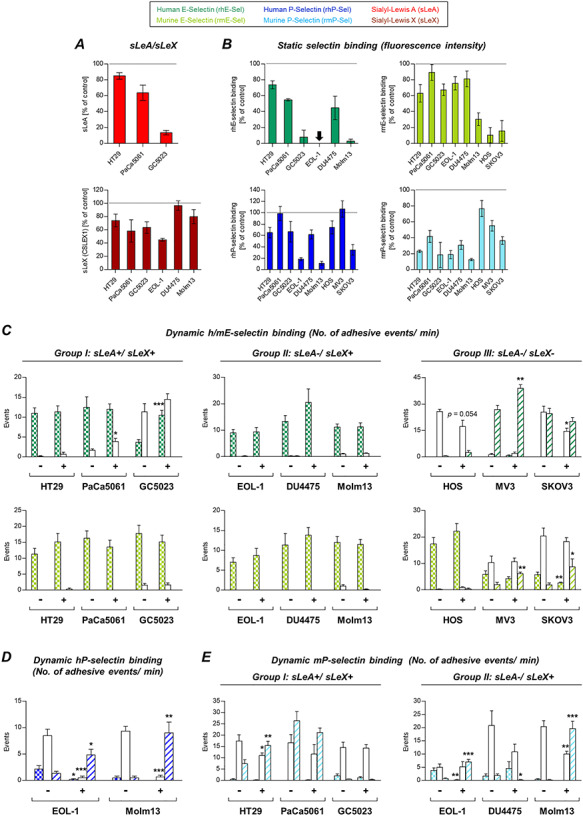
Role of cell surface glycoproteins for tumor cell–selectin interaction. Enzymatic cleavage of cell surface glycoproteins using pronase (*S. griseus*) decreased sLeA and sLeX by a maximum of 50% only except sLeA on GC5023 (~90% reduction) (**A**). The consequences of such treatment for static selectin binding are shown in (**B**). Note the common reduction of static mPSel binding. Dynamic adhesions on E-selectins were only slightly affected (**C**), while the ability of EOL-1 and Molm13 cells to adhere on hPSel and mPSel was strongly decreased (**D** and **E**). In the pronase experiments, we observed less reliable correlation between the treatment effects on static and dynamic mPSel interaction (note the striking effect on static mPSel binding by PaCa5061, GC5023 and DU4475 cells, all of which showed nearly unaltered dynamic adhesion on mPSel). Please see legend to Figure 3 for technical information. This figure is available in black and white in print and in colour at *Glycobiology* online.

Pronase treatment (nonspecifically cleaving glycoproteins from the cell surface) caused weak (~20%) to moderate (~40%) reduction of sLeA expression on HT29 and PaCa5061 cells, respectively, but decreased sLeA on GC5023 cells by ~ 90% (Figure 5A). sLeX expression was weakly to moderately (20–60%) affected by pronase on all cell lines except DU4475, where it was unaltered (Figure 5A). Static binding of hESel decreased upon pronase by 25%, 45% and 55% in case of HT29, PaCa5061 and DU4475 cells, respectively, but was abolished in case of GC5023, EOL-1 and Molm13 cells (Figure 5B). Static binding of mESel remained largely detectable in case of the sLeA/X-positive group as well as EOL-1 and DU4475 cells (reduction by 10–40% only), while it was strikingly diminished in case of Molm13, HOS and SKOV3 cells (Figure 5B). hPSel and mPSel binding was also influenced to variable extent as summarized in Figure 5B.

Concerning the sLeA/X- and sLeX-positive cell lines, the dynamic adhesion on hESel and mESel was not impaired upon pronase treatment (GC5023 cells even showed significantly more firm adhesions). In contrast, sLeA/X-negative cells showed slight but significant changes in the number of adhesions on hESel and mESel (Figure 5C).

Dynamic adhesion on hPSel was strongly affected by pronase treatment in terms of abrogated firm and rolling events. This observation also manifested as considerably increased numbers of tethering interactions (Figure 5D). The used pronase protocol abrogated PSGL-1 expression at the cell surface (Supplementary Figure 1C). Dynamic adhesion on mPSel was affected by pronase in case of some of the tested cell lines as shown in Figure 5E.

### Profiling glycosphingolipid composition after treatment with GalNAc-α-*O*-benzyl

**Fig. 6:**
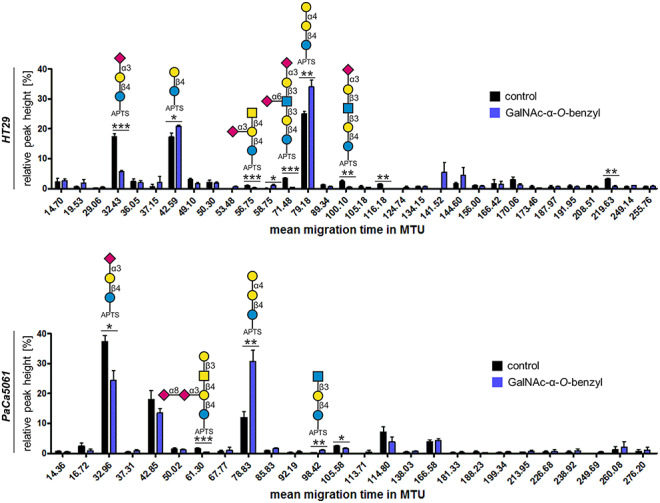
Profiling GSL glycosylation after treatment of tumor cells with GalNAc-α-*O-*benzyl. Treatment of HT29 and PaCa5061 cells with GalNAc-α-*O*-benzyl decreased the abundance of the glycan originating from the ganglioside GM3 (at ~32 MTU) in favor of the glycan globotriaose (Gb3) derived from the globo-series GSL Gb3-Cer (at ~79 MTU). ^*^P ≤ 0.05, ^**^P ≤ 0.01 and ^***^P ≤ 0.001. This figure is available in black and white in print and in colour at *Glycobiology* online.

HT29 and PaCa5061 cells showed both reduced firm adhesions and decreased static binding to hESel after inhibition of *O*-GalNAc-glycosylation, but no such effect was observed after pronase treatment (Figure 4B and C and Figure 5B and C). Therefore, the diversity and abundance of glycosphingolipid (GSL) glycosylation was analyzed for both cell lines before and after treatment with GalNAc-α-*O*-benzyl. By this analysis, we revealed a significant decrease of the glycan derived from the ganglioside GM3 and an increase of globotriaose (Gb3) after GalNAc-α-*O*-benzyl treatment in both cell lines (Figure 6).

### Effects of tunicamycin and swainsonine treatment on tumor–selectin interaction

**Fig. 7:**
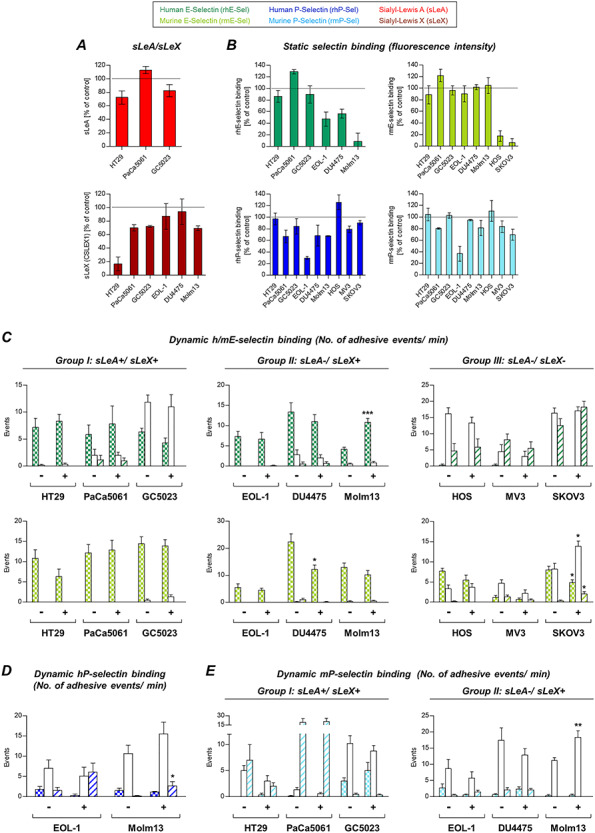
Effects of tunicamycin treatment on tumor cell–selectin interaction. Pharmacologic inhibition of *N*-glycosylation in the ER using tunicamycin only partially reduced sLeA and sLeX expression on the tumor cells; most strikingly, sLeX was reduced by ~ 85% on HT29 cells (**A**). Static selectin binding of sLeA/X-positive cells was mostly unaffected, while strong effects were seen for EOL-1 cells with reduced binding of hESel, hPSel and mPSel and for sLeA/X-negative cells with abolished mESel binding (**B**). Effects of tunicamycin treatment on dynamic adhesion on selectins were rather weak (**C**–**E**). Significant differences were only seen for Molm13 cells on hESel and mPSel (increase) and DU4475 and SKOV3 on mESel (decrease) (**C** and **E**). Please see legend to Figure 3 for technical information. This figure is available in black and white in print and in colour at *Glycobiology* online.

Tunicamycin treatment (impairing *N-*glycosylation in the ER) reduced sLeA expression on HT29 and GC5023 cells (by 30% and 20%, respectively) as well as sLeX on HT29 (by 80%), PaCa5061, GC5023 and Molm13 cells (reduced by 30% each) (Figure 7A). Static hESel binding was convincingly reduced in case of the sLeX-positive group II only (Figure 7B). Static mESel binding only decreased in case of sLeA/X-negative group III cells (Figure 7B). Tunicamycin also altered static hPSel and mPSel binding in some cell lines, but to variable extent as shown in Figure 7B.

The dynamic adhesion to all selectins remained basically unaltered except for a significant reduction of firm adhesions of DU4475 and SKOV3 cells on mESel and a strong increase in adhesions of Molm13 cells on hESel and mPSel (Figure 7C and E).

**Fig. 8:**
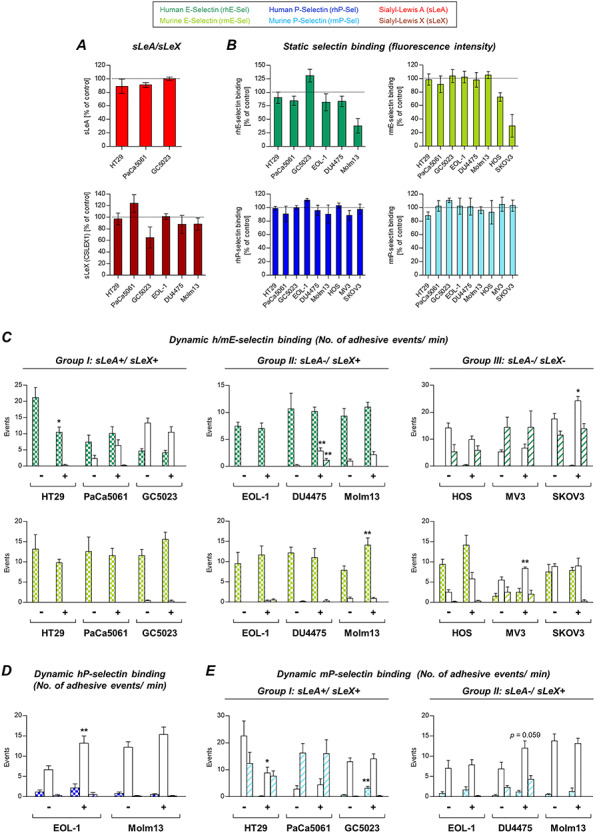
Effects of swainsonine treatment on tumor cell–selectin interaction. Pharmacologic inhibition of *N*-glycosylation in the Golgi using swainsonine had largely no effect on sLeA/X expression and static selectin binding except sLeX expression on GC5023 cells (**A**), static hESel binding by Molm13 cells and static mESel binding by HOS and SKOV3 cells (**B**). Effects on the dynamic adhesion on selectins varied among the cell lines as shown in (**C**–**E**). In particular, adhesions of HT29 cells on hESel and mPSel were decreased (**C** and **E**) while adhesions of SKOV3 cells on hESel, of Molm13 cells on mESel (**C**) and rolling of EOL-1 cells on hPSel were increased (**D**). Please see legend to Figure 3 for technical information. This figure is available in black and white in print and in colour at *Glycobiology* online.

Swainsonine treatment (impairing *N*-glycosylation in the Golgi apparatus) convincingly reduced sLeX expression on GC5023 cells only (by ~ 40%) (Figure 8A). Unexpectedly, however, GC5023 cells showed increased hESel binding after this treatment (by 30%). Despite unaltered sLeX expression, Molm13 cells showed reduced hESel binding after swainsonine treatment (Figure 8B). Static mESel binding was only reduced in case of sLeA/X-negative group III cells (Figure 8B). Static binding of both P-selectins remained unaltered irrespective of the species (Figure 8B).

In the dynamic adhesion experiments on hESel, swainsonine impaired firm adhesion of HT29 cells and improved rolling of SKOV3 cells as well as rolling and tethering of DU4475 cells (Figure 8C). We observed more firm adhesions of Molm13 and more rolling adhesions of MV3 cells on mESel (Figure 8C). The dynamic adhesion on hPSel was only altered in case of EOL-1 cells showing more rolling upon swainsonine treatment (Figure 8D). Dynamic adhesion on mPSel was decreased in case of HT29 cells. In contrast, GC5023 and DU4475 cells showed slightly more firm and rolling adhesions on mPSel, respectively (*P* = 0.059 in case of DU4475, Figure 8E).

See Supplementary Table II for a summary of the observable treatment effects.

## Discussion

To our knowledge, this is the first systematic analysis of human vs. murine E- and P-selectin ligands on human malignant cells under static vs. dynamic binding conditions. Based on our findings, we conclude that E-selectin ligands on human tumor cells are not restricted to sLeA or sLeX (as claimed by several publications St Hill 2011; Reymond et al. 2013; Trinchera et al. 2017). Alternative ligands must exist since the tested sLeA/X-negative cell lines were capable of rolling and tethering on hESel under flow while they were unable to bind hESel in the static experiments. This discrepant functionality of non-canonical E-selectin ligands under static vs*.* dynamic binding conditions strongly supports observations from the 1990s made with leukocytes and leukemic cells (Tiemeyer et al. 1991; Stroud et al. 1996; Handa et al. 1997), where sialosyl-fucosyl poly-*N*-acetyllactosamine without the sLeX epitope was shown to be the physiological selectin ligand under dynamic conditions.

In our study, sLeA/X-negative cells even developed firm adhesions on mESel under flow. This enhanced adhesion strength of tumor cells on mESel as compared to hESel is in line with our second major observation that mESel binds stronger to tumor cells than hESel (irrespective of the tumor cells’ sLeA/X status). Similar observations have already been made with sLeX-coated microspheres and were explained by a larger interdomain angle between the lectin and EGF-like domain and a higher flexibility in key sugar residues of mESel as compared to hESel (Rocheleau et al. 2016). Based on our data, these species-specific differences in the protein structure also strongly affect the binding of real tumor cells to E-selectin. We therefore analyzed whether mESel binds to more carbohydrate structures in a direct binding assay than hESel and found that this is actually the case.

By comparing the different tumor cell groups, our findings overall indicate that sLeA and sLeX are required for static hESel binding. Antibody blockade and tumor cell treatment experiments further support this conclusion. Treatments that effectively reduced sLeA/X expression and static mESel or hESel binding, however, commonly failed to reduce dynamic adhesion on mESel or hESel. Vice versa, some treatments significantly reduced dynamic adhesions on hESel or mESel, but not static hESel or mESel binding. Therefore, tumor cells most probably use different ligands for static vs. dynamic E-selectin binding.

An almost opposing picture emerged for P-selectin: all cells of the sLeA/X-negative group bound P-selectins under static, but not under flow conditions. The “non-canonical” ligands for E- vs. P-selectin are therefore functional under divergent conditions. The static P-selectin ligands on sLeA/X-negative cells were not further analyzed here, but proteoglycans with chondroitin or heparan sulfate glycosaminoglycan chains have been suggested in the past (Koenig et al. 1998; Monzavi-Karbassi et al. 2007). All tested sLeA/X- or sLeX-positive cells showed considerable dynamic adhesion to mPSel indicating that sLeA and sLeX are required for dynamic adhesion of tumor cells to mPSel. However, antibody blockade of sLeA and/or sLeX was not sufficient to impair the dynamic adhesions on mPSel; hence, further ligands together with sLeA and/or sLeX might mediate dynamic adhesion of human tumor cells on mPSel.

The only cell lines with detectable dynamic adhesions on hPSel were the only ones with concurrent expression of PSGL-1 (EOL-1 and Molm13), and these cells also showed a drastic reduction in dynamic and static hPSel binding when PSGL-1 was cleaved by pronase. Therefore, we hypothesized that PSGL-1 represents an important ligand for adhesion on hPSel and that it might be necessary that sLeX is presented on PSGL-1 as the “correct” scaffold. The first hypothesis was verified by the reduction of dynamic adhesions on hPSel after blockade of PSGL-1, which also notably reduced static hPSel binding so that for static and dynamic hPSel binding similar glycoconjugates (certain glycan epitopes on PSGL-1) might be required. Interestingly, the glycan motif is obviously not sLeX alone as shown by antibody blocking of sLeX, which had no effect on dynamic or static hPSel binding. Moreover, PSGL-1 appears to be dispensable for static and dynamic mPSel binding.

Our data overall demonstrate that the static tumor cell/E-selectin interaction could be disturbed by more approaches than the dynamic tumor cell/E-selectin interaction. As mentioned, this observation might be explained by different ligands that are functional under static vs. dynamic conditions (Tiemeyer et al. 1991; Stroud et al. 1996; Handa et al. 1997), but also by the fact that the selectin–ligand bond becomes longer lived at higher mechanical forces (Thomas 2008). This so-called catch bond effect would also explain why several treatments that were sufficient to reduce static E-selectin binding failed to decrease dynamic adhesion. In contrast, effective inhibition of static P-selectin binding frequently coincided with disturbed dynamic adhesion on P-selectin suggesting that catch bonds might strikingly promote E-selectin/ligand interaction, but to lesser extent P-selectin/ligand interaction.

In case of HT29 and PaCa5061 cells, we observed that GalNAc-α-*O*-benzyl reduced dynamic and static interaction with hESel, while non-selective enzymatic cleavage of glycoproteins using pronase did not or only partially disturb the interaction of these cells with hESel. Based on this discrepancy, we hypothesized that inhibition of *O*-GalNAc-glycosylation might have secondary effects on GSL synthesis. GSLs have already been described as potential ligands for E-selectin constituting more than 60% of E-selectin ligands on human neutrophils (Nimrichter et al. 2008). Therefore, we analyzed changes in the GSL composition of HT29 and PaCa5061 cells upon treatment with GalNAc-α-*O*-benzyl. Both cell lines showed a consistent increase of globotriaose (Gb3) accompanied by a decrease of GM3 ganglioside. Interestingly, gangliosides have already been described as E-selectin ligands on human breast cancer cells (Shirure et al. 2011). However, the precise link between *O*-GalNAc-glycosylation and GSL synthesis as well as the role of GM3 gangliosides as putative E-selectin ligands remain to be determined in the future.

The present approaches using blocking antibodies, inhibitors and enzymes aimed at roughly determining general differences between the selectin ligands, but were neither really specific nor able to fully reach the desired effects. Therefore, to further dissect the tumor cell/selectin interaction, more specific approaches such as genetic manipulation of genes codifying glycosyltransferases (e.g., UGCG) might be useful in the future (Mereiter, Martins, et al. 2019). Nevertheless, this study demonstrates underestimated differences in the functionality of static vs. dynamic selectin ligands on solid and hematologic human malignancies with important implications for metastasis research. Likewise, unexpected species-specific differences exist in the ligands for human vs. murine selectins on human tumor cells strongly encouraging the development of immunodeficient mice with humanized E- and P-selectins to enable more clinically relevant metastasis studies in vivo.

## Materials and methods

### Cell culture

Human colorectal cancer cells HT29 and human breast cancer cells DU4475 were purchased from ECACC (Porton Down, UK). The human eosinophilic leukemia cell line EOL-1 was purchased from DSMZ (Braunschweig, Germany) and the human ovarian cancer cell line SKOV3 was from ATCC (Manassas, USA). HOS osteosarcoma cells, Molm13 acute myeloid leukemia cells and MV3 melanoma cells (all human) were kindly provided by the Departments of Pediatric Hematology and Oncology, Oncology and Dermatology, respectively (all University Medical Center Hamburg-Eppendorf, UKE). The human pancreatic cancer cell line PaCa5061 was provided by the Department of General, Visceral and Thoracic Surgery at UKE (Kalinina et al. 2010). The human gastric cancer cell line GC5023 was newly established in the framework of this study (see the next paragraph).

All aforementioned cell lines, except SKOV3, were grown in RPMI-1640 medium with 2 mM L-glutamine, supplemented with 10% fetal calf serum (FCS) and 1% penicillin (50 U/mL) and streptomycin (50 μg/mL) (all from Thermo Fisher Scientific, Waltham, USA), at 37°C with 95% H_2_O-saturated atmosphere and 5% CO_2_. SKOV3 cells were cultured in McCoy’s 5A medium containing 10% FCS, 2 mM L-glutamine and 1% penicillin/streptomycin (P/S) (all from Thermo Fisher Scientific).

### Development and characterization of a novel human gastric cancer cell line

The primary tumor tissue was taken from a 57-year-old female patient who underwent total gastrectomy for advanced gastric adenocarcinoma at UKE. The histopathological examination of the surgical specimen confirmed a low-differentiated adenocarcinoma of the cardia, which was staged pT3, pN2 (10/27), M1 and G3. Following surgery, the patient died 3 weeks after surgery without having received any chemotherapy. A written informed consent of the patient for removal of tissue samples for research purposes was obtained prior to surgery.

Establishment of primary gastric cancer cells GC5023 was performed as previously reported (Kalinina et al. 2010). In short, the tumor tissue was minced into small fragments (~1 mm) and tumor fragments were enzymatically disaggregated using Collagenase IV (0.5%; Sigma-Aldrich, Steinheim, Germany). After incubation for 45 min on a rotary shaker at 37°C, the solution was centrifuged at 700 g for 5 min. The pellet was washed twice in RPMI media (Invitrogen, Carlsbad, USA) followed by resuspension in complete cell culture medium (TUM). The cells were transferred to collagen-coated cell culture flasks (Becton Dickinson, Franklin Lakes, USA) and incubated under standard conditions. TUM medium was prepared as follows: RPMI 1640-Glutamax (Invitrogen) was supplemented with 10% FCS, 1% penicillin/streptomycin, 0.1 mg/mL gentamycin (Biochrom, Germany), 50 nmol/mL human transferrin (Sigma-Aldrich), 0.01 μg/mL recombinant human EGF (PeproTech, Hamburg, Germany) and 0.01 μg/mL human basic FGF (PeproTech). The resultant primary cell line was cultured as monolayer cells in 25–75 cm^2^ flasks, routinely subcultivated by trypsinization and maintained in TUM media. At this time, GC5023 cells underwent less than 50 passages.

### Laminar flow adhesion assay

The dynamic adhesion of human tumor cells to selectins under physiological flow conditions was analyzed at a shear stress of 0.25 dyn/cm^2^ as previously described and illustrated in the box in Figure 1 (Richter et al. 2011). Data were acquired and evaluated with CapImage software (version 8.6, Dr Heinrich Zeintl, Heidelberg, Germany). The adhesive events were distinguished into firm adhesion, rolling and tethering.

### Flow cytometry analysis

The expression of sLeA and sLeX on tumor cells and static binding of recombinant human and murine E- and P-selectins by the tumor cells was assessed by flow cytometry as described before (Wicklein et al. 2013; Lange et al. 2014). Fluorescent mAbs against sLeA (anti-CA19-9) and sLeX (anti-CD15s) were from Novus Biologicals (NBP2-54349AF488) and BD Bioscience (#563528), respectively (final concentration: 1 μg/mL). Fluorescence-labeled mouse IgM served as isotype control (BioLegend, #401617). All flow-cytometric measurements were carried out after marking the cells dead or alive with propidium iodide. Data analysis was performed with FCS Express 4 Flow software (De Novo Software, Los Angeles, CA).

### E-selectin glycan-binding assay

As described previously (Nollau et al. 2013), glycoconjugates (Lectinity, Moscow, Russia) were immobilized in duplicates on flat-bottom MaxiSorp 96-well plates (Thermo Fisher Scientific/Nunc, Rockford, IL) in 100 μL of PBS. Plates were blocked using carbo-free blocking solution (Biozol, Eching, Germany). About 10 μg/mL of recombinant chimeric h vs. m E-selectin/IgG1-Fc constructs (R&D Systems) were precomplexed with biotin-conjugated anti-human IgG (Sigma-Aldrich) and subsequently complexed with streptavidin–HRP (Thermo Fischer Scientific, #21126). Complexes were incubated for 2 h at room temperature, washed three times with TSM buffer (20 mM Tris/HCl [pH 7.4], 150 mM NaCl, 2 mM MgCl_2_ and 1 mM CaCl_2_) in the presence of 0.1% Tween-20. ABTS solution (Roche, Mannheim, Germany) was added as a substrate according to the instructions of the manufacturer. Absorbance was measured at 405 nm on a microplate reader (Tecan, Mannedorf, Switzerland).

### Cell treatment

To generally classify which molecules on the tumor cells mediate static vs. dynamic interaction with human vs. murine E- and P-selectins, the tumor cells were firstly pretreated with nonfluorescent antibodies against sLeA (abcam, #ab3982) and sLeX (BD Bioscience, #551344). Preliminary experiments with the cell line HT29 revealed a dose-dependent effect of the used anti-sLeA/X antibodies on static selectin binding (antibody range: 2–40 μg/mL). Therefore, the highest tested concentration (40 μg/mL) was used for subsequent experiments. Mouse IgM (Dako, #X0942) served as isotype control. Anti-PSGL-1-APC was from eBioscience (#17–1629-41), and blockade of P-selectin binding was made with this mAb at 2 μg/mL. Mouse IgG2a-APC from R&D Systems (#IC003A) was used as isotype control. For all blocking experiment, cells were incubated with relevant antibodies for 30 min onice.

Next, we used enzymatic (neuraminidase and pronase), chemical (GalNAc-α-*O*-benzyl) or pharmacological (tunicamycin and swainsonine) treatments to alter the carbohydrate composition on the cell surface of vital cancer cells. For each of the following treatment protocols, adverse effects on cell viability could be excluded by propidium iodide uptake analyses using flow cytometry (not shown).

Neuraminidase (from *Vibrio cholerae*, Roche) was used at 10 mU/mL in serum-free medium for 1 h under standard culture conditions (Gebauer et al. 2013; Wicklein et al. 2013) to cleave terminal sialic acid-containing sugar residues. Glycoproteins were nonspecifically cleaved by using 1 mg/mL pronase (a broadly active mixture of proteases from *Streptomyces griseus*, Roche) for 45 min at 37°C under serum-free conditions (Kobzdej et al. 2002; Mondal et al. 2016). For the inhibition of *O*-GalNAc-glycosylation, cancer cells were treated with 0.6 mg/mL (2 mM) GalNAc-α-*O*-benzyl (Sigma-Aldrich) in FCS-containing culture medium for 72 h under standard conditions (Huang et al. 1992; Byrd et al. 1995; Huet et al. 1998; Zhang et al. 2014). The *N*-glycosylation of glycoproteins was inhibited by using 3 μg/mL (3.6 μM) tunicamycin (from *Streptomyces sp*., Sigma-Aldrich) for 24 h (Dufour et al. 2017) (blocking *N*-glycosylation in the ER; solvent control: DMSO) or 0.36 μg/mL (2 μM) synthetic swainsonine (Sigma-Aldrich) for 72 h (blocking *N*-glycosylation in the Golgi; solvent control: methanol).

### Glycolipid analysis

For glycolipid analysis, HT29 and PaCa5061 cells were cultivated in the presence or absence of GalNAc-α-*O*-benzyl as described above and equal cell numbers harvested for glycolipid extraction. Glycolipid extraction and subsequent xCGE-LIF analysis was performed as previously described by Rossdam *et al.* (Rossdam et al. 2019). Briefly, the cells were lysed by sonication and glycolipids were extracted in chloroform/methanol (1:2 (v/v)). After removal of cell debris and proteins by centrifugation, the extraction was repeated using chloroform/methanol (1:1 (v/v)) and chloroform/methanol (2:1 (v/v)). The extracts were pooled and further purified on a Chromabond® C_18_ ec polypropylene column (Macherey-Nagel, Germany). Glycans were released from glycolipids using LudgerZyme ceramide glycanase (CGase) from *Hirudo medicinalis* (Ludger, UK) and fluorescently labeled with 8-aminopyrene-1,3,6-trisulfonic acid (APTS, Sigma-Aldrich). Released and labeled glycans were subsequently analyzed by multiplexed capillary gel electrophoresis coupled to laser-induced fluorescence detection (xCGE-LIF) using a remodeled ABI PRISM® 3100-*Avant* Genetic Analyzer (Thermo Fisher Scientific). Data were further processed with GeneMapper™ Software v3.7.

### Statistics

Data were analyzed using GraphPad Prism software (version 5.03, GraphPad Software, Inc., La Jolla, USA) and are presented as means ± SD of the mean fluorescent intensity (flow cytometry) or adhesive events (flow adhesion assay). Comparisons between groups (treated vs. untreated cancer cells) in the flow adhesion assay were evaluated by Student’s *t*-test. Significance levels were defined as follows: ^*^P ≤ 0.05, ^**^P ≤ 0.01 and ^***^P ≤ 0.001.

## Acknowledgements

The authors would like to thank Dr. Jasmin Wellbrock (Research Department of Oncology, University Medical Center Hamburg-Eppendorf) for kindly providing the Molm13 cell line and Christine Knies for excellent technical assistance.

## Funding

German Research Foundation grant to T.L. [LA3373/6-1]; Erich-and-Gertrud-Roggenbuck Foundation grant to T.L.

## Ethics approval and consent to participate

This study describes the establishment of a novel human gastric cancer cell line (GC5023) from a gastrectomy specimen. A written informed consent of the patient for removal of tissue samples for research purposes was obtained prior to surgery and the local ethics committee approved this study (Ethik-Kommission der Ärztekammer Hamburg).

## Consent for publication

Not applicable.

## Availability of data and materials

The datasets used and/or analyzed during the current study are available from the corresponding author on reasonable request.

## Competing interests

The authors declare that they have no competing interests.

## Authors’ contributions

S.S., H.M., V.L., C.R. and G.W.E. collected and analyzed the data. D.W., F.F.R.B. and T.L. interpreted the data. D.W., U.S. and T.L. designed the study. C.G. established and characterized the novel human gastric cancer cell line GC5023 used in this study. C.W., U.S. and T.L. provided resources and supervised the study. S.S. and T.L. were major contributors in writing the manuscript. D.W. and U.S. revised the manuscript. All authors read and approved the final manuscript.

## Supplementary Material

Supplementary_data_cwaa019

## List of abbreviations

GSL, glycosphingolipid; hESel, human E-selectin; hPSel, human P-selectin; LeA, Lewis A; LeB, Lewis B; mESel, murine E-selectin; mPSel, murine P-selectin; sLeA, sialyl-Lewis A; sLeX, sialyl-Lewis X
