# Supplementary material for: Systematic analysis of the human tumor cell binding to human vs. murine E- and P-selectin under static vs. dynamic conditions
Source: Glycobiology. 2020 Feb 27;30(9):695–709. doi: 10.1093/glycob/cwaa019 (PMC7443332; doi:10.1093/glycob/cwaa019)
Supplement: Supplementary_data_cwaa019 [file supplementary_data_cwaa019.docx]

**Supplementary Figure 1. Cell surface expression of PSGL-1 on human cancer cell lines and effect of PSGL-1 blockade on human and murine P-selectin binding.** Among all tested cell lines, only the sLeX-positive cell lines EOL-1 and Molm13 express PSGL‑1 (A) and were the only ones with observable dynamic adhesions on hPSel (see Fig. 1C also). Antibody blockade of PSGL-1 on EOL-1 and Molm13 cells strongly reduces their static binding of hPSel while static mPSel binding remains much less affected (B). In accordance, dynamic adhesions of these cells shifted from firmer (rolling) to looser (tethering) adhesions after PSGL-1 blockade only in case of hPSel, but not mPSel (B). The pronase treatment (strikingly impairing static and dynamic interaction of both cell lines with hPSel and mPSel, see Fig. 5) abolished PSGL-1 expression (C). Black lines in histograms represent isotype controls (A+C) or binding of IgG-Fc (B). Colored lines in histograms of (B) represent hPSel/ mPSel binding after pre-treating the tumor cells with mouse IgG2a (continuous lines) or anti-PSGL-1 (dashed lines). Bars in (B) represent means ± SD of adhesive events per minute from three recordings. **p*≤0.05, ***p*≤0.01, ****p*≤0.001; comparisons were made between isotype- or antibody-treated cells within the subsets of different adhesive interactions (firm, rolling or tethering adhesion).

**
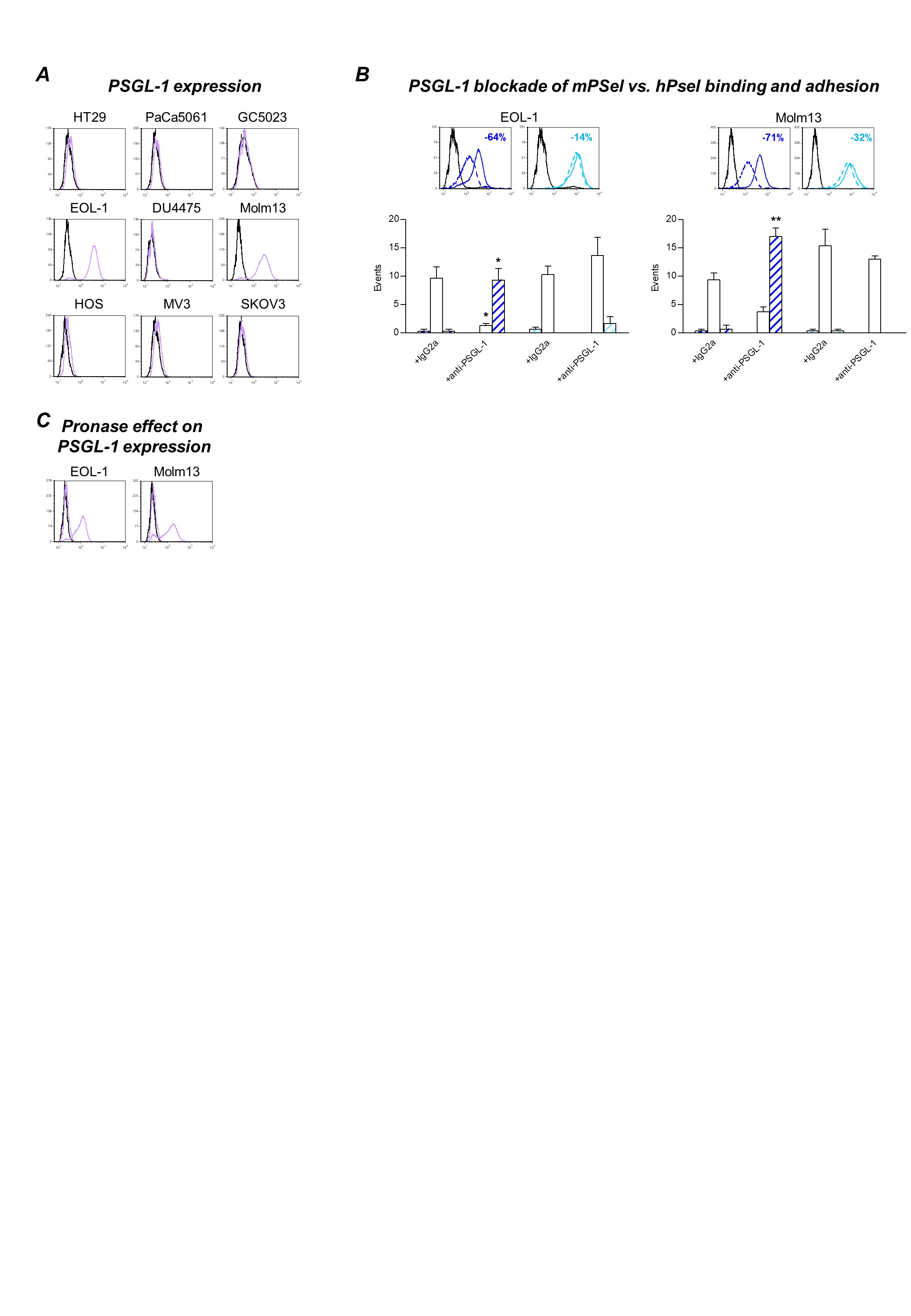
**

**Supplementary Figure 2. Effects of sLeA and/ or sLeX antibody blockade on tumor cell-selectin interaction.** Effects of single and combined blockade of sLeA and/ or sLeX on static and dynamic interaction of sLeA/X-positive tumor cells with selectins are shown in (A+B). Effects of sLeX blockade on static and dynamic interaction of sLeX-positive tumor cells with selectins are shown in (C+D). Of note, blockade of sLeA on sLeA/X-positive cells drastically reduced hESel binding while blocking sLeX had almost no effect (A). Additive effects of combined sLeA/X blockade were only seen with GC5023 cells (A). Static mESel binding was mostly unaffected by antibody blockade and binding of hPSel and mPSel was only partially and variably affected among the cell lines (A). sLeA and/ or sLeX antibody blockade did not affect dynamic adhesion of sLeA/X-positive cells on the different selectins except the adhesions of GC5023 cells on hESel and of PaCa5061 cells on hESel (B). sLeX blockade on sLeX-positive cells commonly sharply decreased hESel binding while only Molm13 cells also showed a decrease in mESel binding (C). Static binding of P-selectin remained largely unaltered upon sLeX blockade except ~35% reduction of mPSel binding by EOL-1 and Molm13 cells (C). The dynamic adhesion of sLeX-positive cells to selectins was not affected by sLeX blockade except the decrease of firm adhesion of DU4475 cells on hESel (D). Bars in (B) and (D) represent means ± SD of adhesive events per minute from three recordings; **p* ≤ 0.05, ***p*≤ 0.01, ****p* ≤ 0.001*,* n.s.= not significant; comparisons were made between isotype- or antibody-treated cells within the subsets of different adhesive interactions (firm, rolling or tethering adhesion).

**
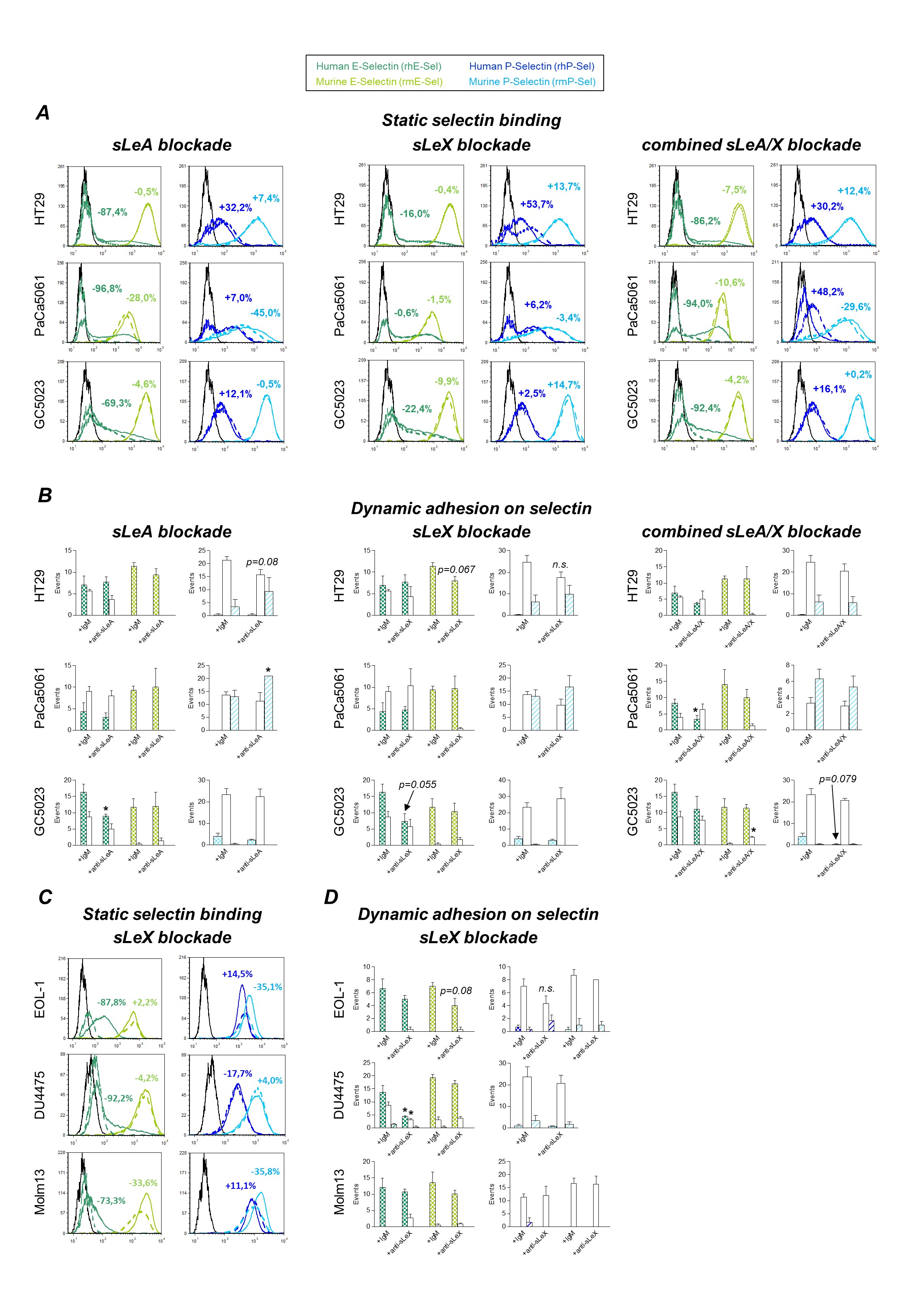
**


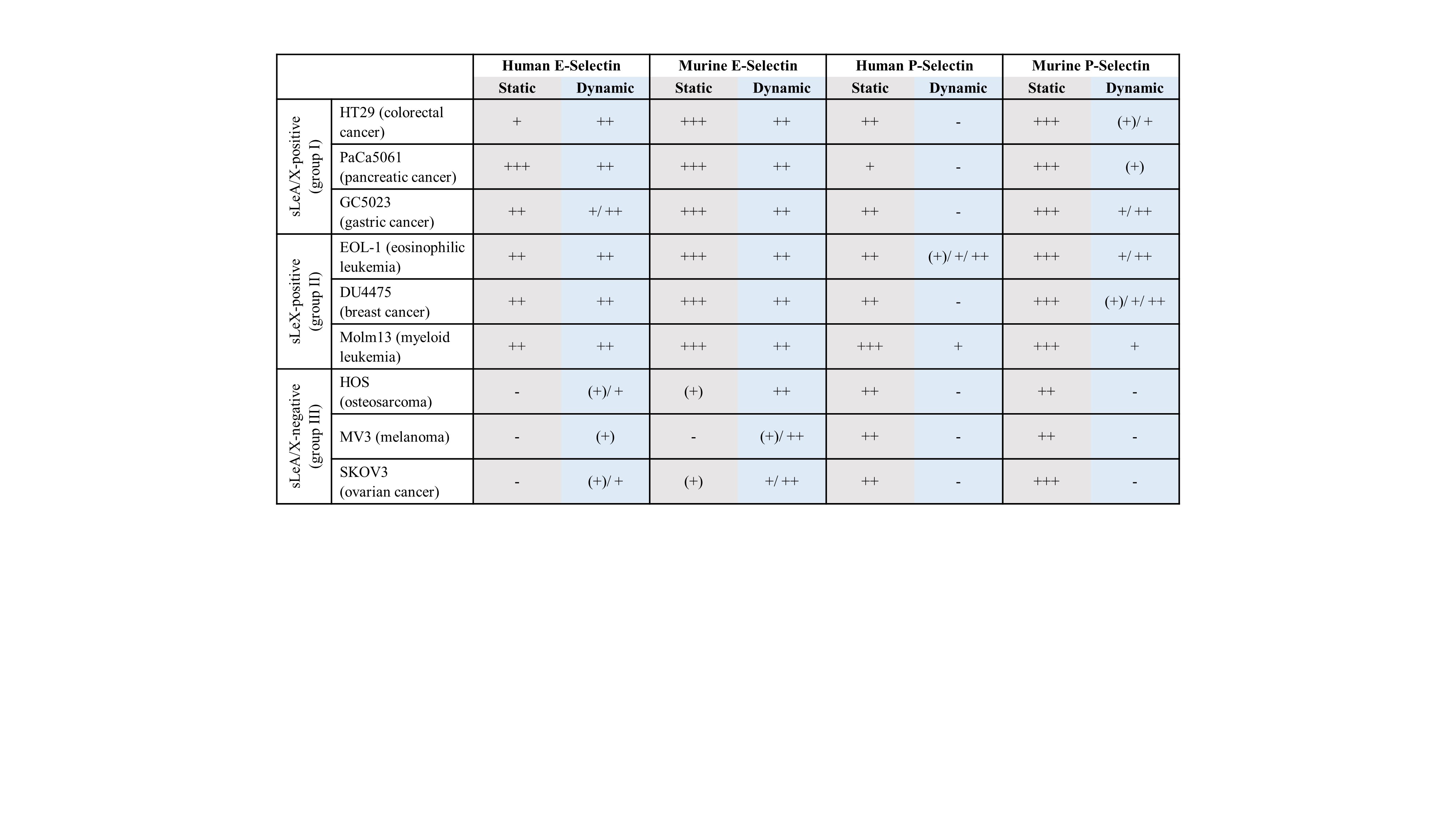
**Supplementary Table I. Summary of static *vs*. dynamic human *vs*. murine E- and P-selectin binding behavior of tumor cell groups I to III (Fig. 1).** In static binding experiments, “-, (+), +, ++, +++” indicate selectin binding strength based on fluorescence intensities. In dynamic adhesion experiments, “-” indicates absence of detectable adhesion, “(+)” indicates tethering adhesions, “+” indicates rolling and “++” firm adhesions.

**
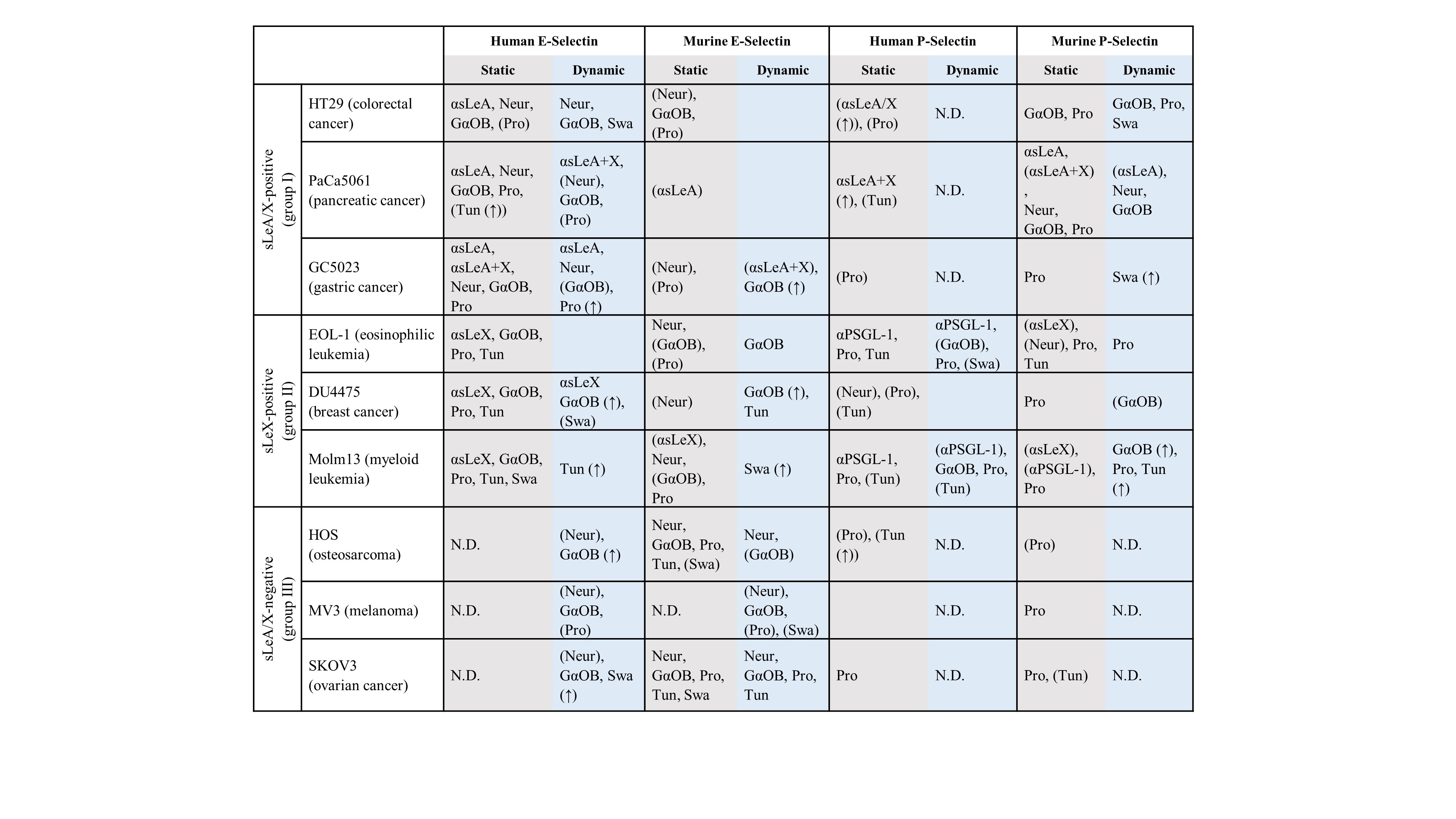
Supplementary Table II.** **Summary of sufficient tumor cell treatments for impairing tumor cell-selectin interaction (unless stated otherwise [↑]) (Fig. 3-5, 7+8 and Suppl. Fig. 1+2).** In static experiments, treatments in brackets indicate rather slight changes (>25%, but <40%). In dynamic experiments, treatments in brackets indicate significant increases in the number of looser adhesions instead of significant decreases of firmer adhesions. αsLeA = sLeA blockade (mAb), αsLeX = sLeX blockade, αsLeA/X = sLeA or sLeX blockade, αsLeA+X = sLeA and X blockade (combined); αPSGL-1 = PSGL-1 blockade (mAb); Neur = Neuraminidase; GαOB = GalNAc-α-O-benzyl; Pro = Pronase; Tun = Tunicamycin; Swa = Swainsonine; N.D. = not detectable.
